# Simultaneous robotic low anterior resection and prostatectomy for adenocarcinoma of rectum and prostate: initial case report

**DOI:** 10.1186/s40064-016-3456-y

**Published:** 2016-10-12

**Authors:** Myungchan Park, Seong Cheol Kim, Jae-Seung Chung, Sang Hyun Park, Seok San Park, Sung Jin Oh, Donghoon Lee, Koon Ho Rha, Cheol Kyu Oh

**Affiliations:** 1 Department of Urology, Haeundae Paik Hospital, Inje University College of Medicine, 248, Jwadongsunhwan-ro, Haeundae-gu, Busan, Korea; 2 Department of Surgery, Haeundae Paik Hospital, Inje University College of Medicine, Busan, Korea; 3 Department of Convergence Medical Science, Institute of Health Sciences, School of Medicine, Gyeongsang National University, Jinju, Korea; 4 Department of Urology, Yonsei University College of Medicine, Seoul, Korea

## Abstract

**Background:**

We report a case of synchronous rectal and prostate cancer treated successfully with simultaneous da Vinci robotic-assisted low anterior resection of the rectum and robotic-assisted laparoscopic radical prostatectomy to address both cancers.

**Case presentation:**

Recently, minimally invasive surgical techniques using da Vinci robot^®^ system (Intuitive Surgical, Sunnyvale, USA) were introduced as curative surgical modality of prostate and rectal malignancies. Herein, we report an initial case of simultaneous robotic low anterior resection and robotic prostatectomy for adenocarcinoma of rectum and prostate sharing a considerable number of port sites.

**Conclusion:**

Simultaneous robotic-assisted low anterior resection could be performed with robotic-assisted radical prostatectomy safely and effectively in synchronous rectal and prostate cancer.

## Introduction

Primary tumors of prostate and rectum are uncommon and have been increasing in incidence worldwide. Some have been diagnosed simultaneously (Baur et al. 1997) and some have invaded each other (Osunkoya et al. 2007; Corral et al. 2000). However, there are several concerns about the two major operations underwent at the same time such as perioperative complications and oncologic outcomes. Although Klee and Grmoljez (1999) reported three patients successfully treated for simultaneous prostate and rectal cancers by radical retropubic prostatectomy and rectal resection at the same operation, there was a slight complication including an early postoperative small bowel obstruction and ischemic colostomy in one patient that required a return to surgery for adhesiolysis and colostomy revision. Another patient developed strictures of the rectal and bladder anastomosis.

Recently, minimally invasive surgical techniques using da Vinci robot^®^ system (Intuitive Surgical, Sunnyvale, USA) were introduced as curative surgical modality of prostate and rectal malignancies. Even though, better surgical outcomes were reported compared with other modalities (Baik et al. 2009; Hohwu et al. 2009), the concept of minimal invasive surgery may be diminished in the simultaneous performance of two major operations. The authors report an initial case of simultaneous robotic low anterior resection (LAR) and robotic prostatectomy for adenocarcinoma of rectum and prostate sharing a considerable number of port sites.

## Case report

A 64-year old man with newly diagnosed rectal cancer was also found to have concurrent prostate cancer. The rigid proctoscopy showed that the tumor location was 10 cm above from the anal verge and biopsies revealed moderately differentiated adenocarcinoma of the rectum. A computed tomography (CT) of the abdomen and pelvis demonstrated annular wall thickening and perirectal fat infiltration but no evidence of distant metastases. Prostate biopsy was performed 3 days after the rectal cancer diagnosis and confirmed prostate adenocarcinoma with Gleason score of 7 (3 + 4) involving 50, 70, and 5 % of each core from the medial right prostate lobe. MRI of the pelvis demonstrated low signal intensity in T2 W images at the rectum and the right posterior portion of the prostate. A whole body bone scan confirmed the absence of skeletal metastatic disease. To address both cancers, simultaneous daVinci robot-assisted LAR and robot-assisted laparoscopic radical prostatectomy were performed.

## Surgical techniques

**Fig. 1:**
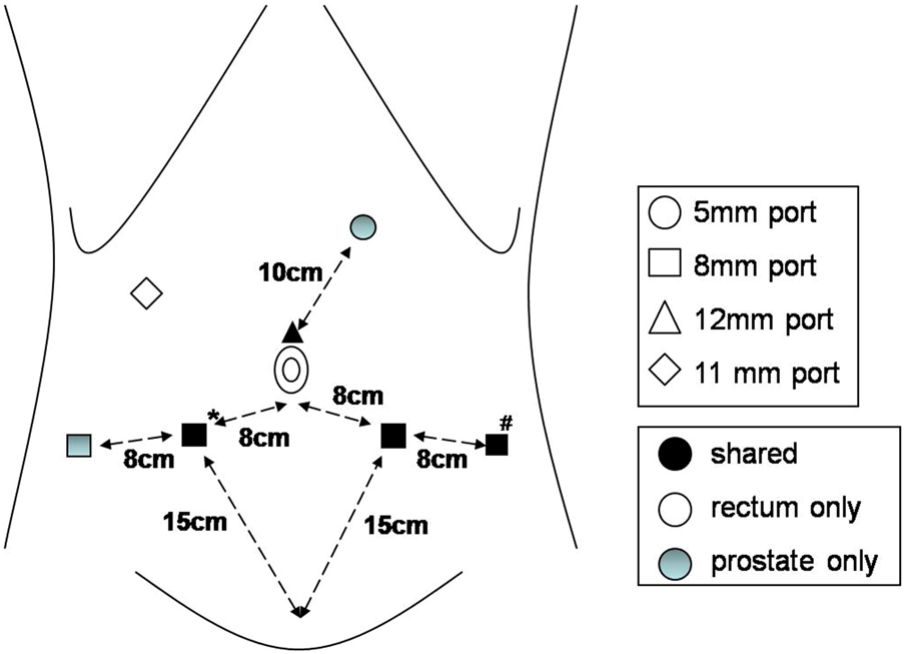
Port sites of daVinci^®^ robot-assisted low anterior resection of the rectum and laparoscopic radical prostatectomy. *Asterisk* Right lateral 8-mm port below the umbilicus which was used to 12-mm trocar for introduction of endo-GIA during robotic assisted low anterior resection. For robot-assisted laparoscopic radical prostatectomy, 8-mm working arm was reinserted with secure suture to prevent CO2 leakage. *Hash* The extraction site of both specimens which was closed once after robotic assisted LAR and 12-mm trocar was replaced for robot-assisted laparoscopic radical prostatectomy

daVinci^®^ robot-assisted LAR was performed first. After induction of general anesthesia, the patient was placed in a modified lithotomy position with the legs apart in a 30° Trendelenburg position and a 15° tilt to the right. A 12-mm trocar was placed through an incision just above the umbilicus after achieving pneumoperitoneum. A 30° standard 12-mm robotic laparoscope was inserted through this trocar. The first 8-mm daVinci^®^ trocar was placed in the mid-point on the line between camera port and the right anterior superior iliac supine. The second and third 8-mm daVinci^®^ trocars were inserted at the one-thirds and the two-thirds points on the line between the camera port and the left anterior superior iliac supine. These four trocars were inserted for the robotic arms which include one camera holding arm and three operating arms. The 11-mm trocar was placed in the right midabdomen cephalad and lateral to the umbilicus in the anterior axillary line to allow assistant access for mobilization of the left colon (Fig. 1).

**Fig. 2:**
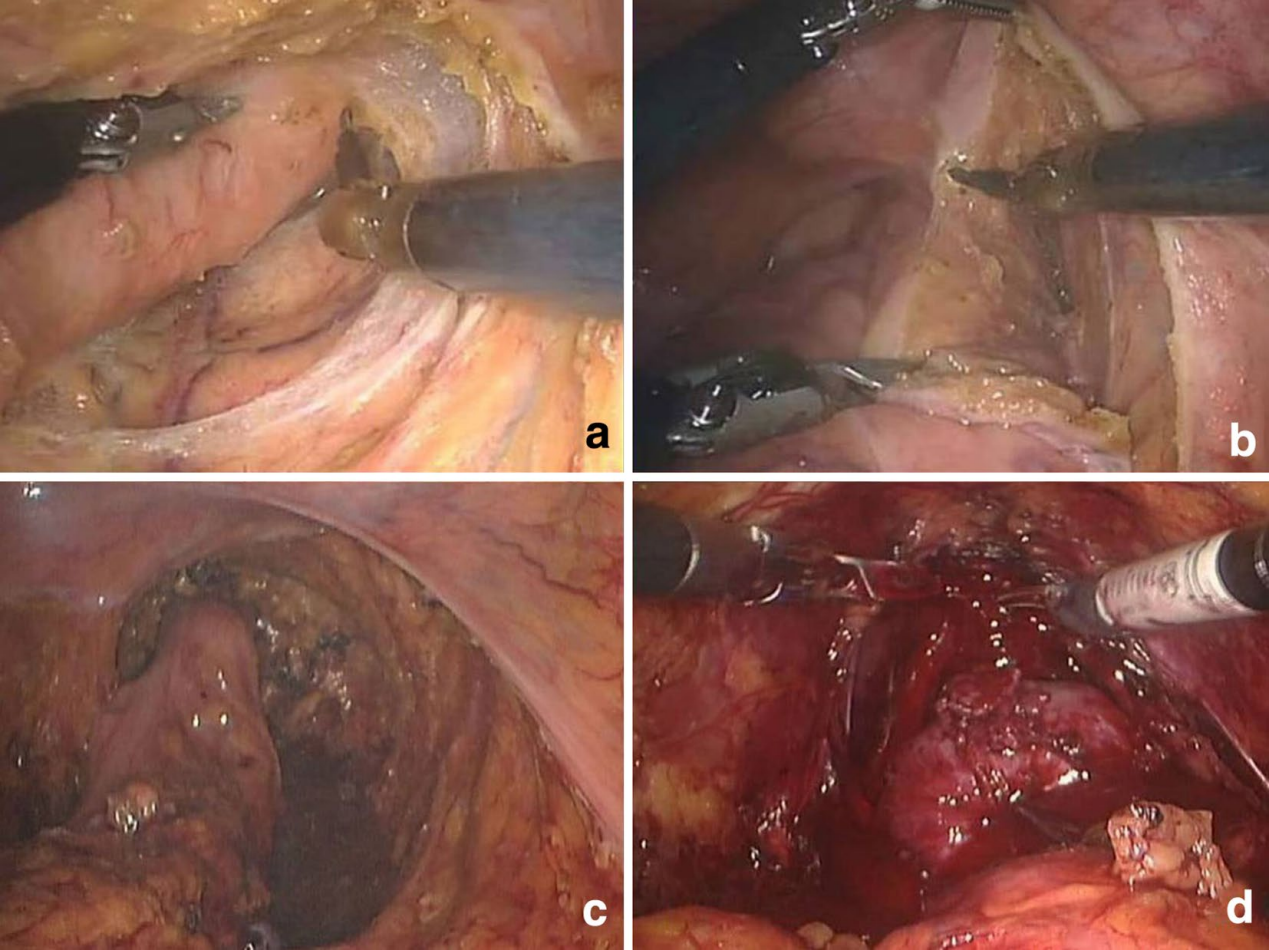
Surgical procedures of the daVinci robot-assisted low anterior resection of the rectum and laparoscopic radical prostatectomy. **a** Posterior dissection in the pelvic cavity using four robotic arms, **b** anterior dissection in the pelvic cavity using four robotic arms, **c** after daVinci robot-assisted low anterior resection of the rectum, **d** daVinci^®^ robot-assist laparoscopic radical prostatectomy

The robot was then positioned between the legs for pelvic dissection. Rectal dissection in the mesorectal plane proceeded in front of the levator ani muscle, using the Cautery spatula in accordance with the total mesorectal excision (TME) principles. At the level of the fourth sacral vertebra, the rectosacral ligament was divided sharply to avoid avulsion of the presacral fascia and the fascia propria of the rectum (Fig. 2a). Anterior dissection was performed last. Anterior elevation of the prostate was provided by the left second robotic arm using the Cadiere grasper. Counter traction was provided by left first robotic arm using the precise™ Bipolar grasper (Fig. 2b). During the procedure, the pelvic autonomic nerve plexus was carefully preserved.

After the mesorectum was dissected up to the rectal wall, the robotic instrument was disengaged. The right 8-mm daVinci^®^ trocar was changed to 12 mm trocar for introduction of the endo-GIA stapler which was used to divide the rectum. The rectum was extracted through the left second 8-mm da Vinci^®^ trocar. An end-to-end anastomosis (EEA) anvil was then inserted in the proximal colon after resection of the specimen extracorporeally and secured with a purse-string suture. The colon was then placed back into the abdomen and the enlarged port site was closed. Pneumoperitoneum was restored, and an EEA stapler was used to create an end-to-end anastomosis. The anastomosis was tested with air insufflation (Fig. 2c).

For robot-assisted laparoscopic radical prostatectomy, the specimen extraction site was closed tightly and a 12-mm trocar was reinserted for use of the laparoscopic grasper. The 8-mm working ports on both sides were also reinserted. Then two additional ports were placed (Fig. 1). The third robotic arm was inserted in the new port at the far right. The 5-mm trocar for suction was placed 10 cm from the camera port towards the left costal margin. Peritoneoscopy was carried out with a 0° laparoscope.

After carrying out bilateral pelvic lymph node dissection, fat overlying the prostate, the endopelvic fascia and the prostatovesical junction were cleared away. The superficial branch of the dorsal vein was coagulated with the bipolar forceps. Incision of the anterior bladder neck exposed the Foley catheter. This was further incised with tailoring according to the presence of medial, lateral, or intravesical projecting lobes of prostate. The posterior bladder neck was also dissected with the ureteral orifice carefully avoided to prevent injury. After transverse incision of the fascia, the vas deferens and the seminal vesicles became visible. Posterior dissection of the prostate was performed previously during LAR. Prostatic pedicles were controlled by with hemoclips with care to preserve the neurovascular bundles. Apical dissection and transection of the urethra were next performed (Fig. 2d). The specimen was then placed in an entrapment bag and temporarily placed away from the operating field. Rocco stitch was done with 3-0 Vicryl. A double-armed suture was prepared by tying the tails of two 3-0 Monocryl (17 + 17 cm) with UR6 needle to perform a running anastomosis using single knot-suturing technique starting from the posterior. A 16 Fr. silastic Foley catheter was introduced, and the balloon was inflated to 5 cc. The anterior urethrovesical anastomosis sutures were then placed completing the anastomosis. No flap was used between two anastomotic sites, and complications such as anastomotic leakage or fistula were not observed during the follow-up period. The resected prostate was extracted through the reopened left 12-mm port site. Estimated blood loss was approximately 350 cc and the total operative time was 360 min. There was no injury during the operation. For prevention of organ injury or additional urine diversion, insertion of ureteral stent or suprapubic cystostomy was not performed during this simultaneous robotic surgery.

## Results

Final pathology revealed that rectal cancer invaded the perirectal fat tissue (pT3) and showed safety margin status. Furthermore, there was no metastasis in 12 regional lymph nodes (pN0) and lymphovascular invasion. The pathologic stage was T3N0M0, stage IIA. The specimen of prostate revealing adenocarcinoma with a Gleason score of 7 (4 + 3) had safety margin status. 17 regional lymph nodes were achieved and there was no metastasis in there. On the second post operative day, bowel activity was already detected. Foley catheter was removed 7 days after surgery. The patient was discharged postoperative day 10 without any complications. Before the operation, the patient’s serum prostate-specific antigen (PSA) level was 4.18 ng/mL (normal range 0.0–4.0 ng/mL), whereas the PSA level reached nadir (<0.01 ng/mL) 2 months after the surgery. MRI of the pelvis performed after 8 months of the operation revealed no local recurrence at the bed of the prostate and rectum. The patient recovered continence 5 months after surgery. The patients could perform sexual intercourse using PED5 inhibitor (Viagra 100 mg) 12 months after the surgery. There was no evidence of recurrence or metastasis of cancer during the 5-year follow-up period.

## Discussion

daVinci^®^ robot-assisted LAR and robot-assisted laparoscopic radical prostatectomy has been performed individually worldwide as surgical modalities for tumors of the rectum and the prostate due to less invasiveness and better perioperative outcomes. They have also been performed safely in our institution (Baik et al. 2008; Chung et al. 2011).

Klee et al. excellently reported the efficiency of simultaneous radical retropubic prostatectomy and rectal resection at a single operative setting (Klee and Grmoljez 1999). We, however, accomplished this method of simultaneous treatment with the assistance of the da Vinci^®^ robot system which we believe allows higher precision and effectiveness in conducting combined radical prostatectomy and rectal resection. As two surgical teams shared the four port sites, we could perform both operations less invasively without disturbing each operative strategy. We could share the port located in left lower abdomen, which was used for extraction site of both specimens, without CO2 leakage.

There are several concerns about radical prostatectomy and LAR of the rectum, such as cost-effectiveness (Lotan et al. 2004) and post-operative urinary incontinence (Potter and Partin 1999). Compared to a non-robot-assisted radical prostatectomy, we consider robot-assisted laparoscopic radical prostatectomy as a step towards technical improvement in the operating room that facilitates surgeons to accomplish improved surgical outcomes and an ameliorated postoperative recovery (Ficarra et al. 2009). In addition, robotic-assisted LAR (Baik 2008) also has technical feasibility and safety in the rectal surgery. Therefore, we think that simultaneous robotic-assisted LAR could be performed with robotic-assisted radical prostatectomy safely and effectively in synchronous rectal and prostate cancer.

## Conclusion

When the patient with synchronous rectal and prostate cancer became a surgical candidate, concurrent robot-assisted low anterior resection and radical prostatectomy would be safe and effective. We accomplished this method of simultaneous treatment with the assistance of the da Vinci^®^ robot system which we believe allows higher precision and effectiveness in conducting combined radical prostatectomy and rectal resection. As two surgical teams shared the four port sites, we could perform both operations less invasively without disturbing each operative strategy. Therefore, we think that simultaneous robotic-assisted LAR could be performed with robotic-assisted radical prostatectomy safely and effectively in synchronous rectal and prostate cancer.
